# Enhancing Cardiovascular Risk Prediction: Development of an Advanced Xgboost Model with Hospital-Level Random Effects

**DOI:** 10.3390/bioengineering11101039

**Published:** 2024-10-18

**Authors:** Tim Dong, Iyabosola Busola Oronti, Shubhra Sinha, Alberto Freitas, Bing Zhai, Jeremy Chan, Daniel P. Fudulu, Massimo Caputo, Gianni D. Angelini

**Affiliations:** 1 Bristol Heart Institute, Translational Health Sciences, University of Bristol, Bristol BS2 8HW, UK; 2 Statistics and Risk Unit (AS&RU), Department of Statistics, School of Engineering, University of Warwick, Coventry CV4 7AL, UK; 3 Faculty of Medicine, University of Porto, 4200-319 Porto, Portugal; 4 School of Computing Science, Northumbria University, Newcastle upon Tyne NE1 8ST, UK

**Keywords:** machine learning, AI, random effects, cardiovascular medicine, risk prediction, expectation–maximization, xgboost

## Abstract

Background: Ensemble tree-based models such as Xgboost are highly prognostic in cardiovascular medicine, as measured by the Clinical Effectiveness Metric (CEM). However, their ability to handle correlated data, such as hospital-level effects, is limited. Objectives: The aim of this work is to develop a binary-outcome mixed-effects Xgboost (BME) model that integrates random effects at the hospital level. To ascertain how well the model handles correlated data in cardiovascular outcomes, we aim to assess its performance and compare it to fixed-effects Xgboost and traditional logistic regression models. Methods: A total of 227,087 patients over 17 years of age, undergoing cardiac surgery from 42 UK hospitals between 1 January 2012 and 31 March 2019, were included. The dataset was split into two cohorts: training/validation (n = 157,196; 2012–2016) and holdout (n = 69,891; 2017–2019). The outcome variable was 30-day mortality with hospitals considered as the clustering variable. The logistic regression, mixed-effects logistic regression, Xgboost and binary-outcome mixed-effects Xgboost (BME) were fitted to both standardized and unstandardized datasets across a range of sample sizes and the estimated prediction power metrics were compared to identify the best approach. Results: The exploratory study found high variability in hospital-related mortality across datasets, which supported the adoption of the mixed-effects models. Unstandardized Xgboost BME demonstrated marked improvements in prediction power over the Xgboost model at small sample size ranges, but performance differences decreased as dataset sizes increased. Generalized linear models (glms) and generalized linear mixed-effects models (glmers) followed similar results, with the Xgboost models also excelling at greater sample sizes. Conclusions: These findings suggest that integrating mixed effects into machine learning models can enhance their performance on datasets where the sample size is small.

## 1. Introduction

Ensemble tree-based machine learning models including Xgboost have been found to be highly prognostic in cardiovascular medicine [1]. The algorithm’s performance across various clinically significant metrics has been previously assessed using the Clinical Effectiveness Metric (CEM), a consensus-based measure that includes a set of constituent components [2,3]: Discrimination (AUC [4], F1 score [5]) assesses the model’s ability to distinguish between outcomes, while calibration (1—ECE [6]) ensures that the predicted probabilities accurately represent the true outcomes. Overall accuracy [7] (1—Brier score [8]) evaluates the closeness between predictions and actual results, and clinical utility (net benefit analysis [9]) measures the practical benefit of the model within a clinical setting.

In statistical models, correlation inflates coefficient estimates, resulting in high variability and unstable models [10]. Group levels within the dataset that represent samples from a population or a probability distribution of group levels, i.e., random effects such as cardiac hospitals, could result in the correlation of samples within each group. However, the extent to which ensemble tree machine learning models can deal with such correlation is largely unknown.

Here, a binary-outcome mixed-effects Xgboost (BME) algorithm is developed and evaluated using CEM, incorporating hospitals as the random effects. Scenarios (different sample sizes) under which the model underperforms compared to the fixed-effects Xgboost (no cardiac centre: NC) model without random effects is also shown. Commonly used glmer and glm models were also assessed to see how alternative mixed-effects machine learning models compare with traditional logistic regression-based mixed-effects models.

Although mixed-effects models incorporating random effects have been widely applied using traditional medical statistics approaches such as in linear mixed and generalized linear mixed models, there are fewer studies (see Section Related Work) on integrating mixed effects into the gradient-boosted tree models for binary classification. Specifically, to the best of our knowledge, the development and application of binary-outcome mixed models have been limited to neural network studies only [11,12].

This article is organized as follows: the remainder of Section 1 reviews related work in this area; Section 2 describes the dataset and patient population analyzed, the exploratory data analysis undertaken, the proposed Xgboost BME approach, and provides the validation approach taken; Section 3 illustrates the application of the method on a cardiovascular dataset; Section 4 gives a discussion in the context of other research as well as some clinical relevance of the approach; Section 5 provides potential future work and the limitations of this study; and finally, a conclusion is provided in Section 6.

### Related Work

Ahlem et al. proposed a mixed-effects random forest (MERF) algorithm developed using Expectation–Maximization (EM) to account for random effects in datasets with continuous dependent variables [13]. In a pilot experimental study, we confirmed that MERF should be used for only continuous outcomes and that for the standard random forest there was limited gain in performance when hospital random effects were converted into a high-dimensional set of 0 and 1 vectors and considered as fixed effects [14]. Ng et al. applied the EM approach to determine the gating network’s weights in a mixture-of-expert-based modelling framework for binary mixed-effects models [11]. The approach was useful in that the estimated weights could be obtained directly from the log likelihood and enabled faster convergence. However, the approach was based on neural networks. In addition, Giora et al. developed an approach called linear mixed model neural network (LMMNN) that defined a negative log likelihood for binary outcomes using the Gauss Hermite Quadrature approximation to estimate the random effects as part of a mixed-effects neural network model [12].

In our previous studies [2,3], it was found that combining the metrics covering all four aspects of discrimination, calibration, clinical usefulness and overall accuracy into a single CEM improved the efficiency of cognitive decision-making (according to Miller’s Law [15]) for selecting the optimal ensemble models [3,14]. This approach is useful for providing a consensus metric that enables models to be ranked in scenarios where, for example, one model could outperform another using one metric, but underperform under a different metric. Furthermore, we demonstrated that such a consensus metric could be combined with drill-down analysis to further interpret the models using individual metrics [3]. While AUC does evaluate the diagnostic or predictive performance of a model, it does not directly reflect patient benefit. This is why we had included within the CEM a suite of other metrics, including the decision curve net benefit index, that were found to be clinically pertinent from our prior study [1].

## 2. Methods

### 2.1. Dataset and Patient Population

This study was performed on data from a national cardiac surgery patient registry (details on the dataset can be found within Appendix A: dataset). The registry provides a rich, time-stamped dataset ideal for evaluating the performance of predictive models in clinical settings due to its comprehensive coverage of diverse patient populations and outcomes. It consisted of a total of 227,087 patients over 17 years of age, undergoing cardiac surgery from 42 UK hospitals between 1 January 2012 and 31 March 2019. The dataset was split into two cohorts: training/validation (n = 157,196, 69.2%; 2012–2016) and holdout (n = 69,891, 30.8%; 2017–2019) as per previous studies [2]. The division into training/validation and holdout cohorts follows standard practices in clinical studies to ensure temporal validation and to assess model generalization to future data [1]. The primary outcome of this study was in-hospital 30-day mortality. As clinical machine learning models with relevance to the tabular dataset are more applicable in the scenario of a large number of variables (i.e., high-dimensional) and traditional statistical scores using a small number of variables have already been well studied, this article examined 60 fixed-effects variables and 1 random-effects variable. The set of 60 fixed-effects variables were determined to be clinically relevant upon consultation with two experienced cardiac surgeons. The protocol for this dataset has been described in detail in the experimental pilot study [14]. However, variable selection requires substantial experimentation work, deserving a paper in its own right, and hence was excluded from the scope of this study.

### 2.2. Exploratory Analysis

An exploratory analysis was conducted by visualizing variation in the mortality rate (%) across hospitals in the training set and test set using the previously validated approach from [16] for facilitating comparison of patterns across geographical locations (hospitals in this case). Horizontal dashed lines were added at the y-axis value that matched the mean mortality rate across hospitals for the two respective plots.

### 2.3. Xgboost BME Approach

We define the Xgboost BME as follows:

(1) $$y_{i}=f(X_{i})+Z_{i}a_{i},\;\;a_{i}\;\sim \;N(0,\;\sigma_{a}^{2})$$

where $f\left(X_{i}\right)$ represents the complex non-linear function for the random-effect cluster i of *m* clusters. As in Simchoni et al. [12], $f\left(X_{i}\right)=f_{i}$ will be used interchangeably from here on; $y_{i}={\left[y_{1},\;\ldots ,\;y_{n_{i}}\right]}^{T}$ is the $n_{i}\;x\;1$ vector of responses for the $n_{i}$ observations in cluster i, $X_{i}={[x_{i1},\;\ldots ,\;x_{in_{i}}]\;}^{T}$ is the $n_{i}\;x\;p$ matrix of fixed-effect covariates, $Z_{i}={\left[z_{i1},\;\ldots ,z_{in_{i}}\right]}^{T}$ is the $n_{i}\;x\;q$ matrix of random-effect covariates, and $a_{i}={\left[a_{i1},\;\ldots ,\;a_{iq}\right]}^{T}$ is the *i*th random-effect cluster of the random effect from the q x 1 unknown vector of random effects having clusters i=1,…,m.

Unlike Hajjem et al. [13], the random effects are considered here to encapsulate the variability in the hospitals as well as any sources of unexplained variation that may be associated with different hospitals. In addition, due to the high computational cost in the context of EM, as well as the rationale that Xgboost uses Boosting rather than Bagging as in random forest, the out-of-bag prediction approach in [13] was excluded from the scope of this study. The Gaussian log likelihood (GLL) was used for maximum likelihood estimation (MLE) through EM with Gauss Hermite Quadrature in order to obtain estimates of $f_{i}$ and $\sigma_{a}$. Since no substantial change was observed in the GLL criterion beyond 10 iterations in the pilot experiments and the computational cost of the EM algorithm applied was high, a minimum number of iterations was applied to avoid early stopping. The first iteration was not considered, and the algorithm kept iterating until the absolute change in GLL was less than a given value, such as $10^{-10}$.

Step 0. Set r = 0. Let $a_{i\left(0\right)}$ = 0, $\sigma_{a\left(0\right)}^{2}$ = 1, $y_{i\left(0\right)}^{*}=y_{i}$.

Step 1. Set r = r + 1. Update $y_{i\left(r\right)}^{*},$ $f_{i,}$ $a_{i\left(r\right)}$.

- Build a forest of trees using a standard Xgboost algorithm with $y_{i\left(r\right)}^{*}$ as the training set responses in logit scale and $x_{ij}$ as the corresponding training set of covariates, i = 1,…,m, j = 1, …, $n_{i}$. Since logits of $y_{i\left(r\right)}^{*}$ are continuous and binary classification using Xgboost is considered, the values were converted back to binary labels using median as the threshold. Given the high class imbalance, with the outcome class (mortality) constituting fewer than 3% of data, employing the median as a threshold dynamically modifies the decision boundary to better detect rare positive instances. Since the Xgboost now models only the fixed-effects component of the response, it was necessary to update the hyperparameters. Random stratified 3-fold Grid Search Cross Validation was applied using the training dataset with the same hyperparameter search criteria as that for the Xgboost NC model, similar to previous studies [1,3]. A maximum of 30 combinations was imposed to allow for variability in parameters across iterations.
- Obtain an estimate of $f_{i(r-1)}$ using the training data on Xgboost in logit scale.
- Estimate $a_{i\left(r\right)}$ using ${\hat{y}}_{i(r-1)}$ and $f_{i(r-1)}$ as inputs into the Gauss Hermite Quadrature using an approach similar to Simchoni et al. [12], where ${\hat{y}}_{i(r-1)}=$ ${logit(y}_{i(r-1)}+\varepsilon )$. The number of quadratures was set at 80, as determined through pilot experiments, satisfying k < 2m − 1, where k represents the degree of the polynomial for numerical integration and m is the adjustment parameter, as the number of random effect levels.
- $y_{i\left(r\right)}^{*}$ = ${\hat{y}}_{i(r-1)}$ − $Z_{i}a_{i\left(r\right)}$, i = 1, …, n, where $y_{i\left(r\right)}^{*}$ represents the fixed component of the response and is re-binarized to 0 and 1 using the median of $y_{i\left(r\right)}^{*}$.

According to [17], the numerical approximation is utilized to predict the conditional mean values of the random effects:

(2) $$\begin{array}{cc} E[a_{i\left(r\right)}|y]\; & \approx \int \frac{f_{y|a_{i}}\left(y{|a}_{i}\right)f_{a_{i}}\left(a_{i}\right)}{f_{y}\left(y\right)}da_{i}\\ & \approx \frac{\log\left\{\sum_{k=1}^{K}\exp\left[\sum_{j=1}^{n}\left({\hat{y}}_{i(r)}\left({\bar{f}}_{i}+\sqrt{2}\sigma_{a_{\left(r\right)}}^{2}x_{k}\right)-\log\left(1+e^{\left({\bar{f}}_{i}+\sqrt{2}\sigma_{a_{\left(r\right)}}^{2}x_{k}\right)}\right)\right)\right]\frac{w_{k}}{\sqrt{\pi }}\right\}\;}{\sum_{i=1}^{m}\log\left\{\sum_{k=1}^{K}\exp\left[\sum_{j=1}^{n}\left({\hat{y}}_{i(r)}\left({\bar{f}}_{i}+\sqrt{2}\sigma_{a_{\left(r\right)}}^{2}x_{k}\right)-\log\left(1+e^{\left({\bar{f}}_{i}+\sqrt{2}\sigma_{a_{\left(r\right)}}^{2}x_{k}\right)}\right)\right)\right]\frac{w_{k}}{\sqrt{\pi }}\right\}}, \end{array}$$

where

$$f_{a_{i}}\left(a_{i}\right)\approx \;\frac{w_{k}}{\sqrt{\pi }},$$

$$f_{y}\left(y\right)\;\approx \;\sum_{i=1}^{m}\log\left\{\sum_{k=1}^{K}\exp\left[\sum_{j=1}^{n}\left({\hat{y}}_{i}\left({\bar{f}}_{i}+\sqrt{2}\sigma_{a_{\left(r\right)}}^{2}x_{k}\right)-\log\left(1+e^{\left({\bar{f}}_{i}+\sqrt{2}\sigma_{a_{\left(r\right)}}^{2}x_{k}\right)}\right)\right)\right]\frac{w_{k}}{\sqrt{\pi }}\right\},$$

$f_{y|a_{i}}\left(y{|a}_{i}\right)$ is the conditional density function of mortality given random effects from hospital i and ${\bar{f}}_{i}$ is the mean of estimates from Xgboost on training data for cluster i. $f_{y}\left(y\right)$ is also the GLL.

Step 2. Update $\sigma_{a_{\left(r\right)}}^{2}$ using

(3) $$\begin{array}{cc} \mathrm{Var}(E[y_{ij}|a_{i}]) & =\;\sigma_{a_{\left(r\right)}}^{2}\\ & \approx \;\mathrm{var}({\bar{f}}_{i}+a_{i})\\ & \approx \;\sum_{i=1}^{m}\frac{n_{i}{\left({\bar{y}}_{i}-\bar{y}\right)}^{2}}{m-1}\\ & \approx \;\sum_{i=1}^{m}\frac{n_{i}{\left({({\bar{f}}_{i}}_{\left(r\right)}+a_{i\left(r\right)})-\bar{y}\right)}^{2}}{m-1} \end{array}$$

where ${\bar{y}}_{i}={{\bar{f}}_{i}}_{\left(r\right)}+a_{i\left(r\right)}$ is the empirical average of the predicted response values at RE level i and $\bar{y}$ is the empirical average of the actual response, $y_{i}$, across all RE levels on the logit scale.

Step 3. Keep iterating by repeating steps 1 and 2 until convergence.

We ran the algorithm for 20 iterations and stopped adding additional iterations as there were little change in performance.

According to [12], the likelihood function is as follows:

(4) $$GLL=\;\sum_{i=1}^{m}\log\left\{\sum_{k=1}^{K}\exp\left[\sum_{j=1}^{n}\left(y_{i}\left(f_{i}+\sqrt{2}\sigma_{a_{\left(r\right)}}^{2}x_{k}\right)-\log\left(1+e^{\left(f_{i}+\sqrt{2}\sigma_{a_{\left(r\right)}}^{2}x_{k}\right)}\right)\right)\right]\frac{w_{k}}{\sqrt{\pi }}\right\}$$

### 2.4. Validation Approach

#### 2.4.1. Xgboost BME and NC Variant Models

In order to provide a reliable estimate of model performance and its variability, the geometric mean of the Clinical Effectiveness Metric (CEM) and individual component metrics were evaluated using 1000 bootstraps for the Xgboost BME and NC model variants that had either features that were standardized or unstandardized. The 95% confidence intervals were also calculated from the bootstrap sampling for the CEM.

Using a similar approach, the CEM and its individual components were assessed for the glm and glmer model variants with and without standardization.

#### 2.4.2. Performance by Sample Size

CEM and AUC performances were evaluated against different sample sizes ranging from low (300–1000), medium (2000–10,000) to high (15,000-full sample size), specifically 300, 500, 700, 1000, 2000, 5000, 10,000, 15,000, 15,500 and 157,196. These were evaluated for the two best models from each of the mixed and fixed Xgboost model variants, respectively, i.e., the unstandardized Xgboost BME and standardized Xgboost NC models. In addition, performance was evaluated for the two best models from each of the mixed glmer and fixed glm model variants, i.e., standardized glmer and unstandardized glm models. Log_10_ transform of the sample size was performed along the x-axis of the figures.

#### 2.4.3. Visualization of Parameters

The values of $a_{i}$ are kept in the log-odds space and plotted across the 42 hospitals by their indices across all the sample sizes in the Section 2.4.2. Since $a_{i}$ contains random effects due to both the hospital and any remaining residual error effects, we centred the $a_{i}$ effects by subtracting the mean.

Based on the CEM plot by sample size, the $a_{i}$ across 20 iterations was visualized for the unstandardized Xgboost BME model at a sample size (n = 2000) that showed marked differences between the Xgboost BME and Xgboost NC models. To show the point of convergence, the GLL objective function was plotted across 20 iterations.

#### 2.4.4. Baseline Models

This study consulted with two cardiac surgeons on the most frequently used logistic regression (LR) models used in their clinical studies. It was found that glm and glmer were the most commonly used and they were not interested in further parameter optimization for LR in their studies. As such, these models were included as baseline comparison models.

## 3. Results

### 3.1. Exploratory Analysis

**Figure 1:**
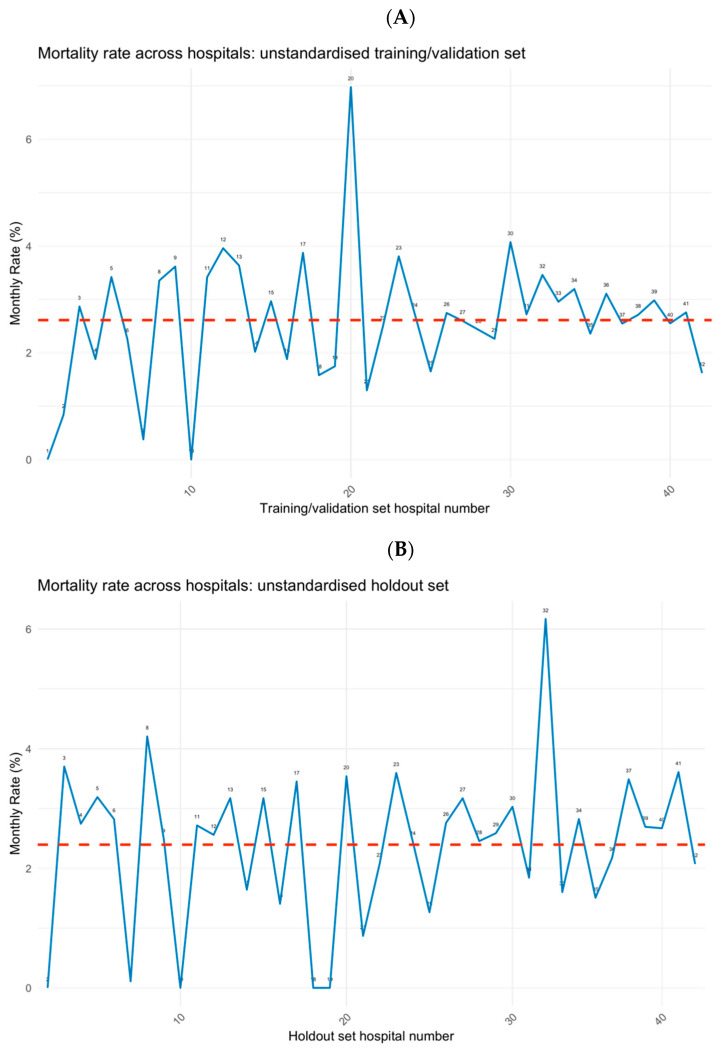
Comparisons of the mortality variation across hospitals in the (**A**) training/validation and (**B**) holdout (test) sets; hospital numbers are shown on the x-axis; the red lines show the average mortality rate across hospitals.

The exploratory analysis showed hospital-related variability in mortality across the training and test datasets. This variability highlights the necessity of accounting for hospital-level effects in predictive modelling, justifying the use of mixed-effects models in this context. Notably, the peak near hospital 20 showed a very large peak in the training set, whilst the peak was diminished in the test set (Figure 1). Conversely, the peak at 32 was diminished in the training set but was magnified in the test set.

### 3.2. Model Validation: Comparison Using All Samples

#### 3.2.1. Xgboost BME and NC Variant Models

The standardized Xgboost NC model demonstrated slightly higher performance (CEM 0.741: 95%CI: 0.7405–0.7411; Table 1) than the other Xgboost model variants when all training data samples were utilized. However, this difference is marginal and may not translate into practical clinical benefits, emphasizing the importance of considering model complexity and interpretability. The performance of unstandardized Xgboost BME and NC did not differ (CEM: 0.740) with overlapping confidence intervals. However, the standardized Xgboost BME model showed the lowest performance (CEM: 0.739, 95%CI: 0.7391–0.7397). There were negligible differences across individual component metrics.

#### 3.2.2. Glmer and Glm Variant Models

The CEM of standardized glmer and unstandardized glm showed a higher magnitude (CEM: 0.719) compared to the other two model variants (CEM: 0.718) due to slightly higher contributions of either AUC or F1 scores, respectively. However, there was very little evidence of the difference being significant across variant models of glmer and glm with confidence intervals overlapping for CEM estimates, ranging from 0.7181 to 0.7189 (Table 2). AUC values were higher for the glmer models (AUC: 0.827) than the glm models (AUC: 0.826), suggesting that remaining differences in CEM across models may be mostly attributed to differences in F1 score.

### 3.3. Performance by Sample Size

#### 3.3.1. Unstandardized Xgboost BME and Standardized Xgboost NC Models

**Figure 2:**
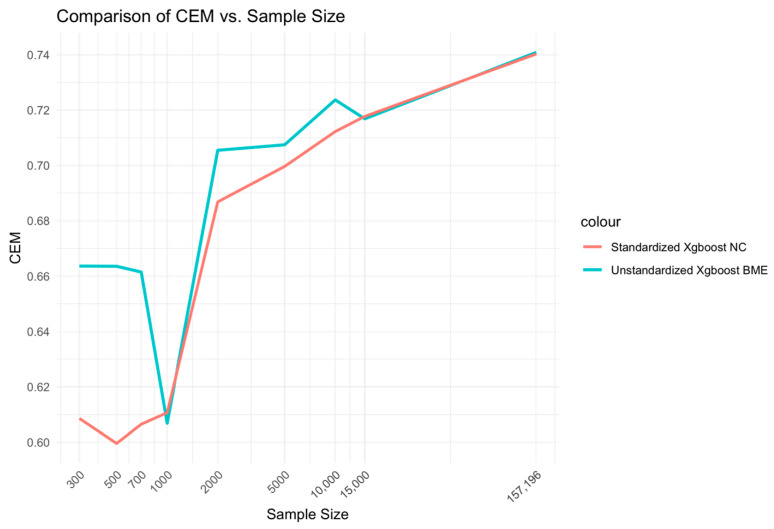
Relationship between sample size and CEM for standardized Xgboost NC and unstandardized Xgboost BME models.

**Figure 3:**
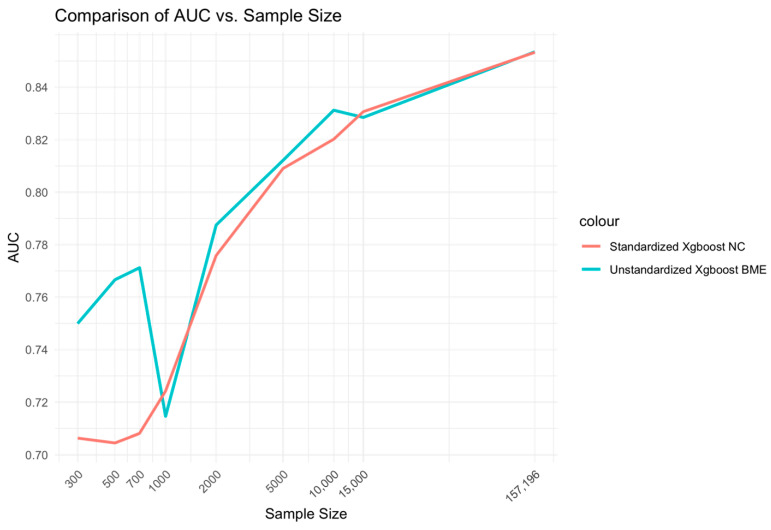
Relationship between sample size and AUC for unstandardized Xgboost BME and standardized Xgboost NC models.

At low sample sizes of 300–1000, the unstandardized Xgboost BME model outperforms the standardized Xgboost NC by a large margin (Figure 2). This relationship holds for medium-range sample sizes, although the size difference is reduced. Beyond n = 15,000, little to no difference is observed across the two models. A similar relationship is observed for AUC (Figure 3). 

#### 3.3.2. Unstandardized Glm and Standardized Glmer Models

**Figure 4:**
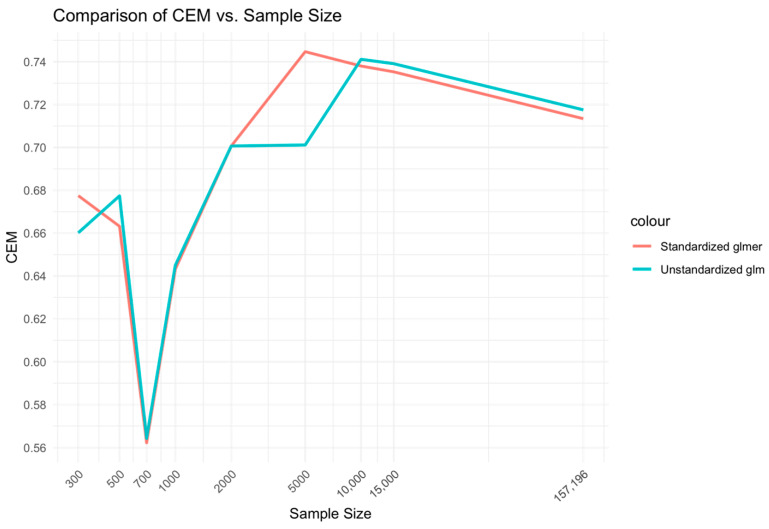
Relationship between sample size and CEM for unstandardized glm and standardized glmer models.

In the comparison between the unstandardized glm and standardized glmer models (Figure 4), a similar relationship was found to the Xgboost BME vs. NC models. That is, the medium range of sample sizes, 2000–10,000, displayed higher CEM performance for the mixed-effects Xgboost BME model compared to the fixed-effects Xgboost NC model. However, differences between the glmer and glm models at low sample sizes of 300–1000 did not demonstrate a marked difference from that observed for the Xgboost model comparisons.

While the glm and glmer models showed higher overall CEM performance compared to the Xgboost models for middle-range sample sizes, the performances of Xgboost BME and NC were higher for large sample ranges. While the Xgboost BME model showed similar performance to the glm and glmer models at low sample ranges, the performance of the Xgboost NC model was substantially lower.

**Figure 5:**
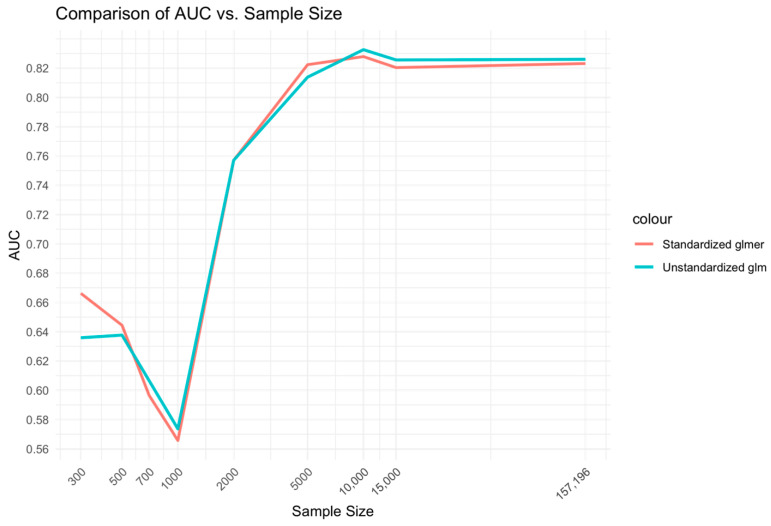
Relationship between sample size and AUC for unstandardized glm and standardized glmer models.

The relationship of sample size to AUC was similar for the logistic regression (glm and glmer) to that of the Xgboost model comparisons but with relative advantage of the glmer over glm at low ranges to medium ranges of sample size being less prominent (Figure 5).

### 3.4. Visualization of Parameters

**Figure 6:**
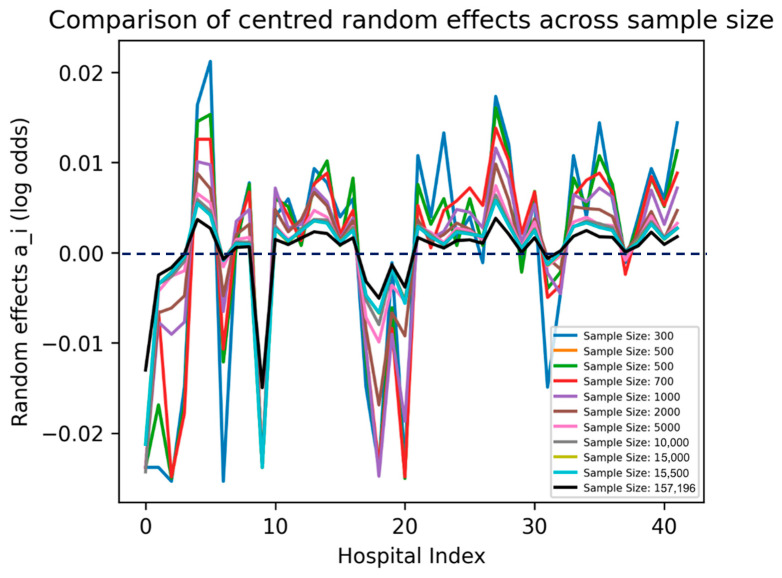
Unstandardized Xgboost BME: random effects (a_i) across hospitals; the line at y = 0 can alternatively be considered as Odds Ratio = 1 if transformed from log odds, i.e., no effect on mortality.

As sample size increased, the magnitude of the random effects decreased (Figure 6). This concurs with earlier results which showed that the effect of the mixed-effects models was larger at low–medium sample ranges compared to high sample ranges. As these random effects relate to the estimates of the model using the training/validation set, a comparison could be made to the mortality rate of hospital 20 in the training set (Figure 1A). It can be seen that the random effects at this point were diminished, suggesting that the high variability of hospital 20 was suppressed. This suppression may be beneficial since in the test set (Figure 1B), the peak at hospital 20 was very small in relation to the training set.

**Figure 7:**
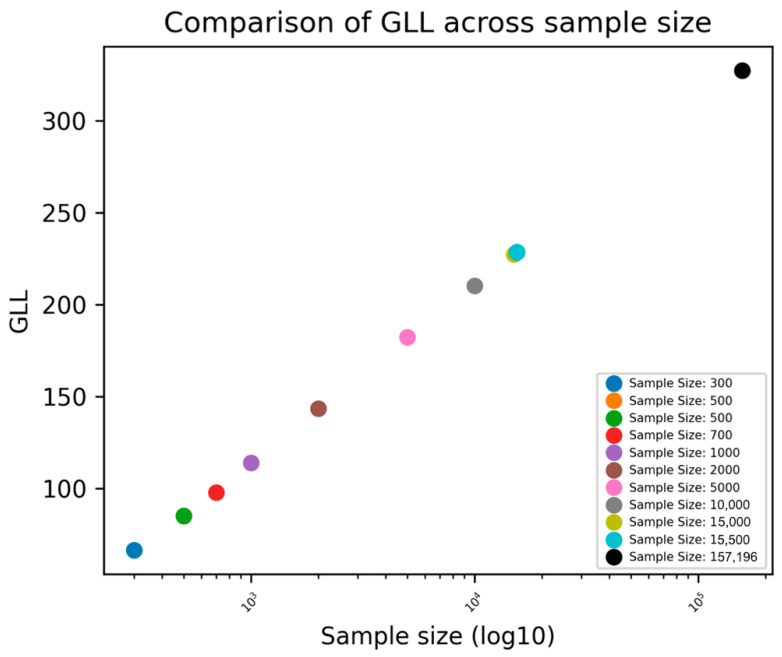
Unstandardized Xgboost BME: GLL across different sample sizes.

The GLL was shown to increase as sample size increased, indicating an improvement in the fit of the model (Figure 7).

## 4. Discussion

In this study, it was found that the performance of mixed-effects machine learning models varied across different sample sizes with the tendency for higher performances in low to medium ranges of samples compared to the fixed-effects models. Whilst these models still demonstrated high performances with large sample sizes, the impact of random effects was diminished. To explore this further, one could consider random effects from an alternative perspective. According to [17], the theoretical conditional mean values of the random effects is as follows:

(5) $$E[a_{i}|y]=\frac{\sigma_{a}^{2}}{\sigma_{a}^{2}+\frac{\sigma^{2}}{n_{i}}}({\bar{y}}_{i}-\mu )$$

where $\sigma_{a}^{2}$ represents the between-cluster (or intercept) variance and $\sigma^{2}$ can be considered the residual variance and μ represents the true population mean of the mortality across all possible cardiac surgery hospitals. According to [17], these three parameters are unknown and hence have been estimated here using machine learning combined with numerical integration approaches. Counter-intuitively, it could be observed that when the sample size is large, individual hospital samples $n_{i}$ will be large, leading to the ratio $\frac{\sigma_{a}^{2}}{\sigma_{a}^{2}+\frac{\sigma^{2}}{n_{i}}}$ tending towards 1. This suggests that instead, the decrease in random effects at large sample sizes is more likely due to the decreased deviation of the hospital-specific mortality rate from the mean mortality rate across hospitals $\left({\bar{y}}_{i}-\mu \right)$ as sample size becomes large. Possible reasons for this could be related to the decreased effects of hospital-specific extreme outliers as the average mortality rate is obtained from an increasingly larger number of patients that dilutes the effects of outliers. This may partly explain why at larger sample sizes, random effects and hence the effect of the hospital on the prediction of mortality are diminished. On the other hand, the average mortality rates of patients in hospitals with low sample sizes may be severely affected by variations in only a few mortalities. The precise modelling of larger variations from extreme outliers at low sample sizes through integrating random effects may also help to partly explain the performance gain observed at low sample sizes.

Specifically, at low sample ranges, the mixed-effects Xgboost BME outperformed the fixed-effects Xgboost NC model by a large margin, potentially enabling Xgboost BME to have more applicability for small datasets. The contrast in performance difference was substantially smaller between the mixed-effects logistic regression glmer and the non-mixed-effects glm, although in these two models the contrast is primarily found in medium-sample-size datasets.

### 4.1. Technical Perspective

The literature review by Peter et al. found that “using machine learning on small size datasets present a problem, because, in general, the ‘power’ of machine learning in recognising patterns is proportional to the size of the dataset, the smaller the dataset, the less powerful and less accurate are the machine learning algorithms [18]”. The challenge is further exacerbated when the clinical outcome is rare, whereby the small dataset may have a non-representative outcome variable frequency. For example, in cardiac surgery where the average mortality rate is often less than 3%, the number of mortalities at the smaller sample size may be difficult to extrapolate. Common approaches for dealing with low sample sizes that have been have been proposed and implemented in the literature include data augmentation through generative adversarial networks (GANs) [19], as well as regularization, an approach that adds additional parameters or constrains to prevent overfitting [20]. These approaches include adding a dropout rate modification to neural networks or defining early stop criteria during training.

While performance was similar between the mixed-effects variant models at low sample ranges, it was found that the mixed-effects Xgboost (BME) model demonstrated higher performance at large sample ranges, while the mixed-effect logistic regression (glmer) showed higher performance at medium sample ranges. This suggests an intricate relationship between sample size and the effectiveness of mixed effects on machine learning models.

The idea of incorporating random effects in tree-based machine learning models has been considered by Ahlem et al. [13]. Given many biological processes that are under study in cardiovascular medicine and beyond, their approach is likely to find application for continuous outcomes whereas the Xgboost BME may be more suited for binary outcomes, for example, whether the patient survives or not or experiences a post-operative complication or not.

Giora et al.’s use of Gauss Hermite Quadrature approximation for approximating the random effects in mixed-effects neural networks for binary dependent variable scenarios provides the basis for extending this approximation approach to other machine learning models such as Xgboost [12]. Their approach made use of the neural network’s inherent capabilities to incorporate the random-effects-based negative log likelihood for binary dependent variables as the loss function. This enabled the neural network’s performance to surpass that of the glmer model.

While Ng et al. used the EM approach to estimate the weights of their MoE model, the method adopted for estimating the likelihood is that of a residual or restricted maximum likelihood (REML) using derivative-based maximization approaches rather than a Gauss Hermite Quadrature-based approach [11]. In addition, their evaluation methods were based on the use of misclassification percentages rather than the CEM and its component metrics.

In an algorithm developed by Lu et al. to handle high-dimensionality datasets, it was found that convergence could occur rapidly in under five iterations [21]. The Xgboost BME algorithm showed similar performance since convergence occurred early rather than late.

The inclusion of hospital IDs as a single fixed-effects variable in the model decreases interpretability by imposing numerical ordering on naturally nominal category values, which is not conceptually meaningful. This method could result in inaccurate readings of the effect estimates since it presupposes an ordinal link between hospital identifiers, which is not the case.

One-hot encoding is an alternative technique for fixed-effects coding that breaks down the hospital variable into a set of binary (0/1) indicators, each of which represents a different hospital. One-hot encoding enables direct comparisons between each hospital and a composite reference group while maintaining some interpretability. However, this strategy still reduces clinical interpretability because it compares to an abstract group without a clear clinical reference, hindering understanding of hospital-specific outcomes. The increased dimensionality expands the model’s degrees of freedom, increasing the danger of overfitting, particularly in models with small sample sizes or significant variability. This can produce unstable estimates, reducing the model’s generalizability and clinical value. Furthermore, the added complexity of numerous hospital-specific parameters presents substantial challenges for clinicians, who may struggle to extract clear, actionable insights from these as separate variables. As a result, despite its statistical precision, this technique ultimately limits practical interpretation in clinical contexts.

The binary-outcome mixed-effects Xgboost (BME) model accounts for random-effect changes in hospital performance while remaining interpretable. This approach allows for an assessment of how much each hospital’s results deviate from the general average after controlling for other factors. By including random intercepts, the model captures hospital-specific variations and quantifies the variance attributable to each hospital, allowing inter-hospital comparisons.

### 4.2. Relevance to Clinical Practice

#### 4.2.1. Cardiac Surgery Perspective

The variation in the cardiac surgery hospital mortality rate is a complex topic that deserves to be discussed. Previous studies have indicated that consultant performance has limited effect on outcomes but that the level of patient comorbidity across the demographics of different hospitals and across different time periods has an effect [22,23]. Although such data were not available to this study, the effect of post-operative critical care provision variations across hospitals, time and age may also have an impact [22]. Nonetheless, variations in age [23] and other important risk factors across time, such as operative urgency, weight of intervention (i.e., complexity of the mix of procedures performed), severity of heart failure (New York Heart Association Functional Classification—NYHA score), level of renal impairment and repeat operations, have been found to have varying effects on outcomes across time in previous studies on both the current and other datasets [2,14].

Random-effects modelling can be applied into day-to-day clinical practice. For instance, several studies have assessed the effects of regional/national level variations in treatment interventions while accounting for patients’ characteristics and their socioeconomic profiles [24,25]. By using a random-effects approach, this can reduce the chance of overfitting that would occur by analyzing individual regions/hospitals separately. Furthermore, integration with machine learning approaches could enhance predictive accuracy while retaining interpretability.

The potential use case of the XGBoost BME model for pediatric congenital heart surgery data is especially relevant considering the challenges of small sample sizes in this clinical context [26]. Paediatric congenital heart surgery frequently involves heterogeneous and complex patients, making linkage across electronic health records and large dataset collections challenging due to the rarity of problems, the wide diagnostic and surgical strategy heterogenicity, and the relatively smaller samples size compared to adult cardiac surgery. Traditional machine learning models may struggle to perform well on these small datasets when the number of covariates is high, resulting in suboptimal predictions and inferences. Hence, these reasons make the development of such ML models a very urgent and required clinical priority in this field.

Subject to ethical approval applications, outcome monitoring after cardiac procedures in congenital heart disease (OMACp) or a similar congenital heart disease dataset could be analyzed [26], as these datasets capture the clinical complications and procedural variances encountered in pediatric patients. Random effects such as the site of catheterization or surgical centre can be integrated into the model to account for inter-site variability, further enhancing the robustness of predictions.

#### 4.2.2. Cardiology Perspective

Random-effects models are reported in the literature to be beneficial for bias reduction through better identification of patient heterogeneity (e.g., patients with different responses to drug treatment) [27]. They may be advantageous for obtaining repeated patient measures [28], improving generalizability [29] and increasing the predictive accuracy of ECG analyses for enhanced patient outcomes. Xgboost BME could also have an application for prediction tasks in heart rate variability (HRV) studies. A large portion of early work carried out in this area (especially for congestive heart failure (CHF)) adopted tree-based algorithms to deploy their models due to the interpretability of these models [29,30,31]. HRV is the time intervals between consecutive heartbeats. In healthy subjects, these time intervals can be highly variable. This is, however, not the case in patients with diseased hearts where HRV measures are depressed. Essentially, higher values of HRV indicate healthier hearts. The presence of random effects in HRV measures can be due to lifestyle factors, individual differences, the types of devices used for HRV measurements, differences in the conditions under which HRV is measured (physical activity, time of day, posture, stress level, age categories, etc.) and variation across different experimental study conditions. Xgboost BME could be used to account for these differences in variability that coexist within different levels of the HRV data hierarchy. HRV measures are obtained from electrocardiogram (ECG) signals, and they exist in the time, frequency and non-linear domains. Xgboost BME could have utility in improving prediction tasks in these domains since ECG signals simulate the presence of random effects across the different domains, thus making more accurate and personalized interpretations possible. Xgboost BME could also enhance the extraction of ECG-related intra-subject correlations that capture individual-specific baseline ECG characteristics, and account for individual variability across multiple sites and devices [32,33].

## 5. Future Work and Limitations

Although Xgboost BME holds potential for improved performance over many of these scenarios, more research is needed to determine how it can be used to better understand data distribution patterns, address sample size issues, interpret complex results, reduce the effect of outliers or influential data points on estimates of heterogeneity, and decrease the computational complexities and explainability associated with large datasets or complex hierarchical structures. This then leads to the question of the efficacy of adopting nested random effects for model improvement. In this scenario, ranges of one grouping variable are completely associated with specific levels of another grouping variable to account for the structure and size of the sample data. Models incorporating this approach have been proposed in the literature to improve the accuracy and interpretability of predictions by capturing variability at different levels of the data hierarchy [34]. The Xgboost algorithm is hierarchical in nature and can naturally handle nested data, but may potentially lead to increased model complexity, making the model too complicated for clinicians to understand. Several ways to address this issue have also been proposed. In the design and deployment of nested random-effects models, strategies focusing on model simplicity (adopting simple models that adequately represent the data and use of appropriate model selection criteria) [35], clarity (defining clear hierarchical structures in the data by combining or collapsing levels and/or evaluating the need for each nesting level) [36,37] and clinical relevance (using visualization and diagnostics tools to assess the distribution of random effects) are recommended [37,38]. Wherever possible, model interpretation is to be prioritized over model fit. Also, when communicating with clinicians, simple technical language and avoidance of statistical jargon are advised when describing the model to help clinicians grasp the impact of variability between different patient groups and to ensure they understand and use the results effectively.

Some existing uses of nested design models in healthcare settings include modelling the correlation between repeated measures taken from the same individual over time in longitudinal studies [39], evaluation of variability in treatment effects in patients nested across several clinical trial centres [40], robust estimation of randomized clinical trial effect sizes through efficient sampling [41] and optimized estimations of the overall effects of study outcomes [42].

Future studies should also consider creating rating scales based on the predicted risk of patients from binary mixed-effects machine learning models. For example, risks can be grouped across different severity of risks: high, medium and low risk. Alternatively, with clinical input, one may create new rating scales for benchmarking the quality of services provided by hospitals and integrate such ratings as standalone or additional random effects [43]. Such rating scales may also be useful in studies involving clinical questionnaires. As the number of hierarchical levels in multi-level random effects increase, future work could also consider the use of confirmatory factor analysis (CFA) to test the most suitable groupings for rating scales for input into mixed-effects ML models [43].

Many of the above-mentioned aspects were out of the scope of this study. However, future work on the Xgboost BME model could incorporate some of the methods and algorithms used in the cited studies.

## 6. Conclusions

In this study, a binary-outcome mixed-effects algorithm for ensemble tree machine learning models has been presented. Performance gains over fixed-effects models and traditional glm/glmer models demonstrated a complex sample-size-dependent relationship that deserves further research in future studies. These findings suggest that integrating mixed effects into machine learning models can enhance their performance on datasets with low sample sizes. However, the specific scenario for such application should be a personalized decision.

## Figures and Tables

**Table 1:** CEM and individual component metrics for Xgboost BME and NC variant models.

Model Category	ECE	AUC	Brier	F1	Net Benefit	CEM	CEM Lower 95% CI	CEM Upper 95% CI
standardized Xgboost BME	0.998	0.854	0.977	0.293	0.908	0.739	0.7391	0.7397
unstandardized Xgboost BME	0.997	0.854	0.977	0.294	0.908	0.740	0.7396	0.7402
standardized Xgboost NC	0.997	0.854	0.977	0.295	0.908	0.741	0.7405	0.7411
unstandardized Xgboost NC	0.997	0.854	0.977	0.293	0.908	0.740	0.7394	0.7400

**Table 2:** CEM and individual component metrics for glmer and glm variant models.

Model Category	ECE	AUC	Brier	F1	Net Benefit	CEM	CEM Lower 95% CI	CEM Upper 95% CI
standardized glmer	0.993	0.827	0.973	0.269	0.889	0.719	0.7182	0.7188
unstandardized glmer	0.993	0.827	0.973	0.269	0.889	0.718	0.7178	0.7184
unstandardized glm	0.994	0.826	0.973	0.270	0.889	0.719	0.7183	0.7189
standardized glm	0.994	0.826	0.973	0.269	0.889	0.718	0.7181	0.7187

## Data Availability

The data underlying this article were provided by NICOR/HQIP under licence/by permission. Data will be shared on request to the corresponding author with permission of NICOR/HQIP.

## Appendix A

Dataset. The analysis was performed using the National Adult Cardiac Surgery Audit (NACSA) dataset, which comprises data prospectively collected by the National Institute for Cardiovascular Outcome Research on all cardiac procedures performed in all National Health Service (NHS) hospital sites and some private hospitals across the UK. The register-based cohort study is part of research approved by the Health Research Authority (HRA) and Health and Care Research Wales, and since the study used de-identified data, a waiver for patients’ consent was waived (HCRW) (IRAS ID: 278171). A total of 227,087 patients over 17 years of age, undergoing cardiac surgery from 42 UK hospitals between 1 January 2012 and 31 March 2019, following the removal of 3930 congenital cardiac surgery cases, 1586 transplant and mechanical support device insertion cases and 3395 procedures missing information on mortality, were included in this analysis. There were 6258 deaths (mortality rate of 2.76%). The primary outcome of this study was in-hospital mortality. Missing and erroneously inputted data in the dataset were cleaned according to the National Adult Cardiac Surgery Audit Registry data pre-processing recommendations; The dataset was split into two cohorts: training/validation (n = 157,196; 2012–2016) and holdout (n = 69,891; 2017–2019) as per previous studies.
